# Supplementary material for: Endonucleosis mediates internalization of cytoplasm into the nucleus
Source: Nat Commun. 2024 Jul 11;15:5843. doi: 10.1038/s41467-024-50259-3 (PMC11239883; doi:10.1038/s41467-024-50259-3)
Supplement: Supplementary file 1 — Supplementary Information [file 41467_2024_50259_MOESM1_ESM.pdf]

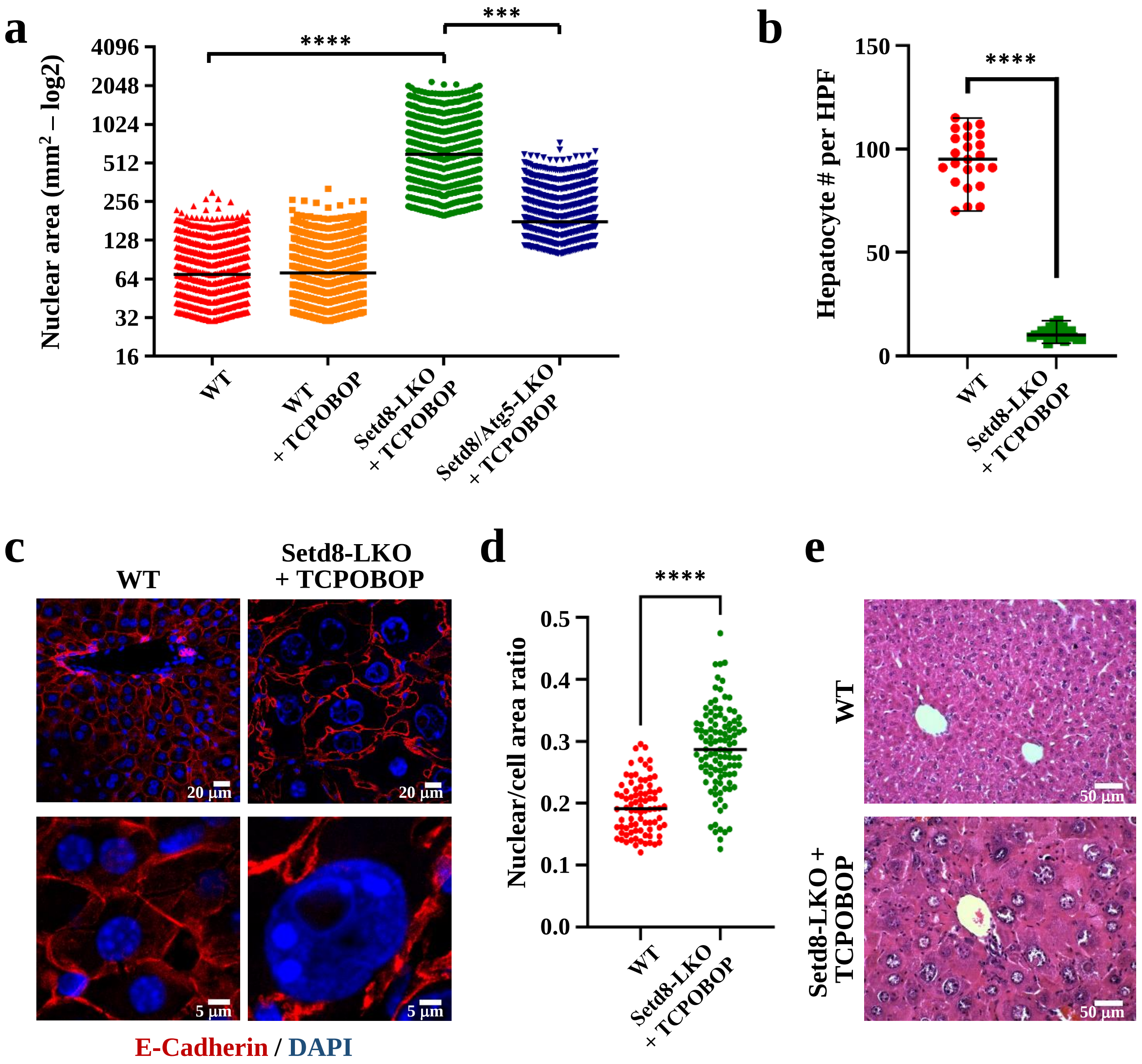

Supplementary Fig. 1

Supplementary Fig. 1

Characterization of cell and nucleus size in hepatocytes displaying endonucleosis.
a, Comparison of the relative nuclear areas. High Content Microscopy measurements were performed in n=4295 nuclei of wild type hepatocytes, n=4476 nuclei of TCPOBOP-treated wild type hepatocytes, n=4095 nuclei of TCPOBOP-treated Setd8-LKO hepatocytes, and n=4091 nuclei of TCPOBOP-treated Setd8/Atg5-LKO hepatocytes. Black horizontal lines indicate median values. Data analysis was performed using one-way ANOVA test. **b,** Quantification of hepatocyte numbers in 23 High Power Fields (HPF) in wild type and TCPOBOP-treated Setd8-LKO livers. Data analysis was performed using Student's *t* test. Mean values and standard errors are indicated. For **a** and **b**, *** p-value <0.001; ****p-value<0.0001.
c, E-Cadherin staining to determine cellular borders and the number of nuclei in hepatocytes. Note the occasional presence of binuclear cells in both wild type and TCPOBOP-treated Setd8-LKO hepatocytes. **d,** Determination of the relative nuclear size compared to total cell areas in n=110 wild type and n=107 TCPOBOP-treated Setd8-LKO hepatocytes. **e,** Hematoxylin-eosin (H&E) staining of liver sections from wild type and TCPOBOP-treated Setd8-LKO mice.
Source data are provided as a Source Data file.

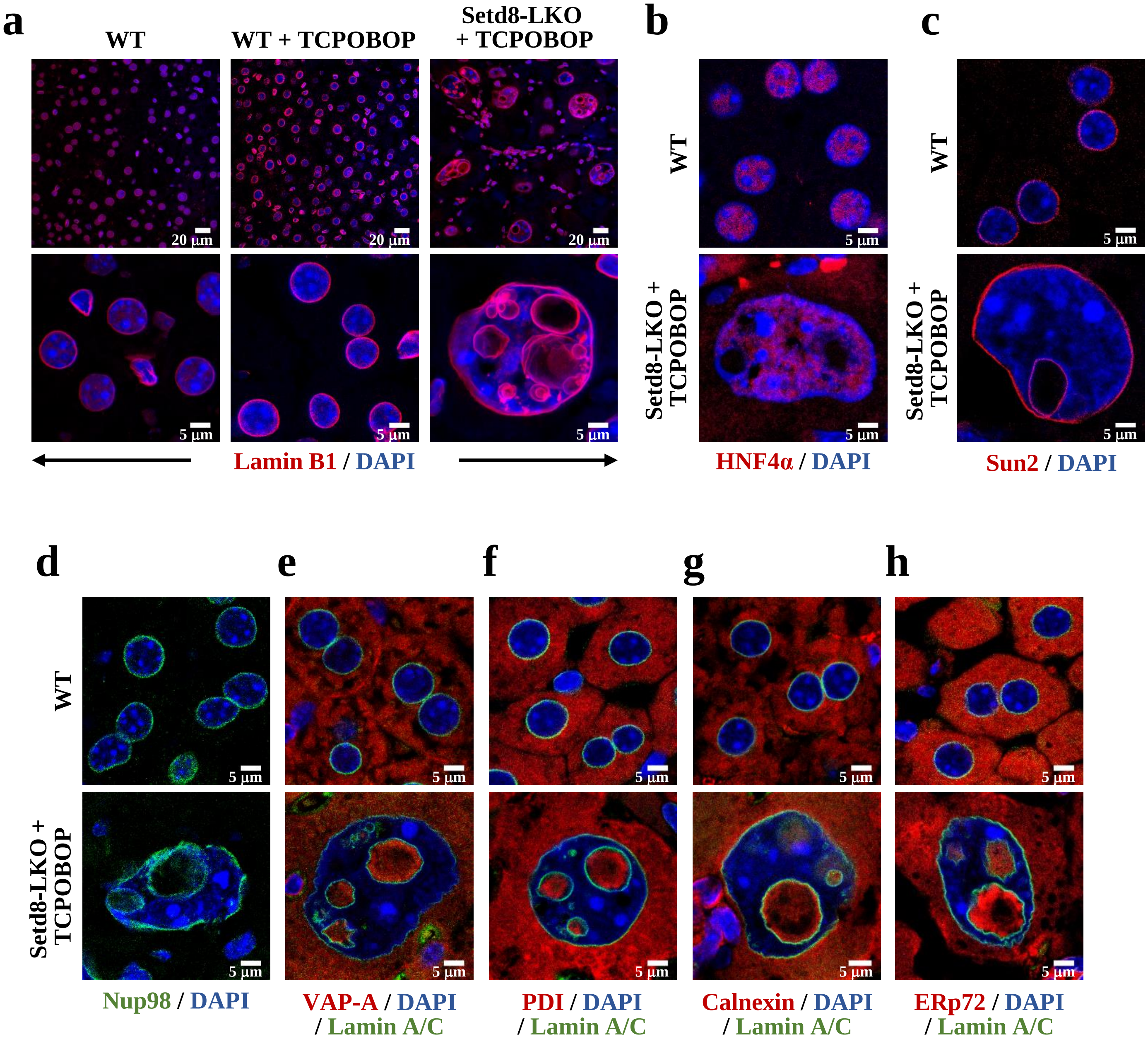

Supplementary Fig. 2

Supplementary Fig. 2

Nuclear envelope composition in hepatocytes displaying endonucleosis.

a, Lamin B1 antibody immunostaining of liver sections from wild type (WT) mice and wild type or Setd8-LKO mice after 24 hours of treatment with TCPOBOP. Fluorescent images of the cells are shown at two different magnifications. **b,** Immunofluorescence detection of the nuclear protein HNF4α. **c-h,** Immunofluorescence detection of the inner nuclear envelope component Sun2 **(c)** the nuclear pore complex component Nup98 **(d)**, the outer nuclear membrane-associated protein VAP-A **(e)**, and the endoplasmic reticulum markers PDI **(f)**, Calnexin **(g)** and ERp72 **(h)**.

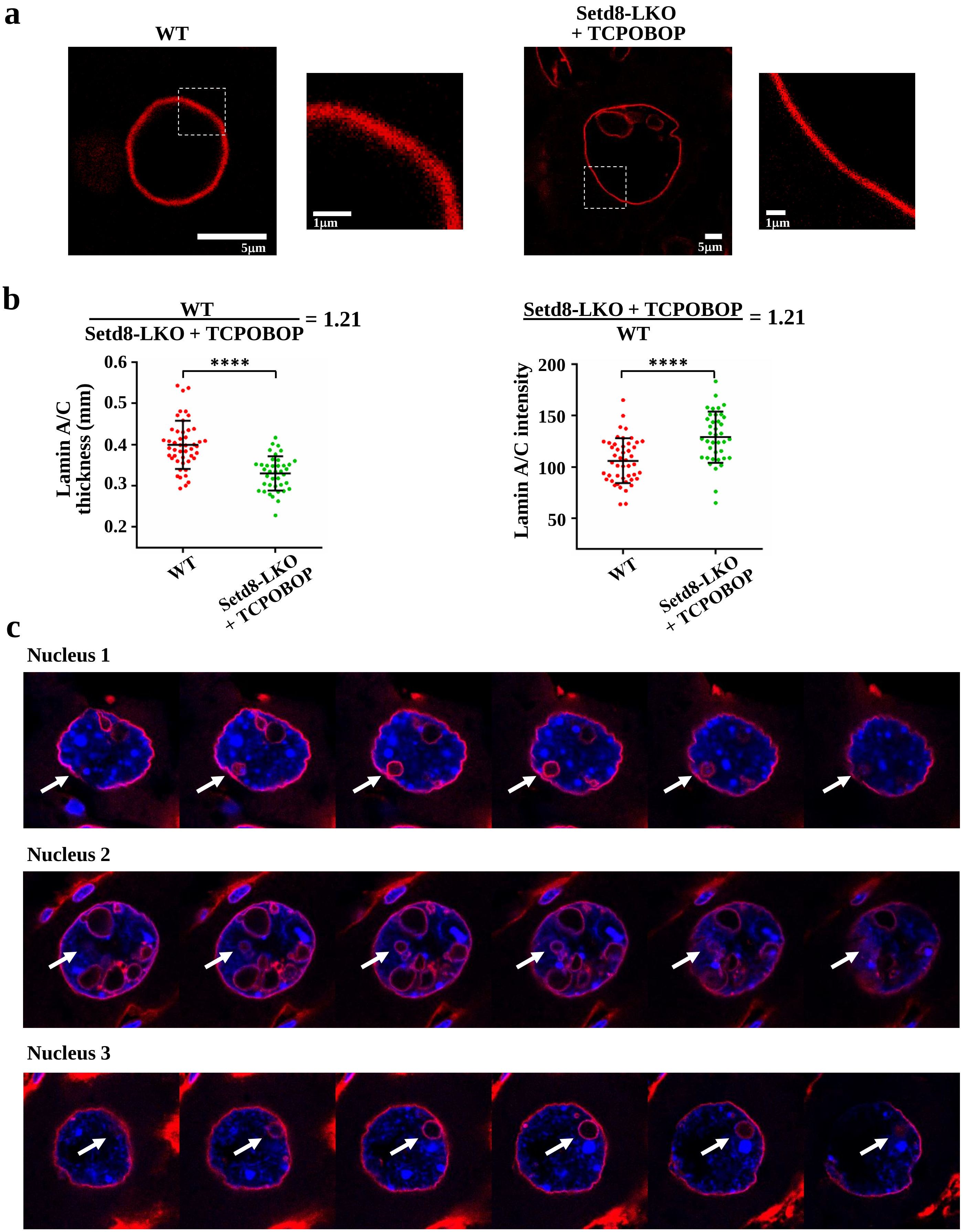

Supplementary Fig. 3

Supplementary Fig. 3

Evaluation of Lamina thickness and the intranuclear positioning of lamin-coated vesicles.
a, Representative close-up images of Lamin A/C-stained nuclear envelope areas from wild type and TCPOBOP-treated Setd8-LKO hepatocytes. **b**, Measurements of the thickness and pixel intensity of multiple individual areas of 47 WT and 38 Setd8-LKO hepatocyte nuclei. The same individual areas were used for both thickness and pixel intensity measurement. Data analysis was performed using Student's *t* test. Data are presented as median values (black line) with SD. ****p-value<0.0001.
Note that, while the spatial distribution of Lamin A/C signal per unit areas were less dispersed (thinner) in Setd8-LKO nuclei, the average pixel densities were proportionally increased in the same areas.
c, Serial confocal z-stack images of nuclei used for 3D reconstruction imaging in Fig. 1b, showing the entirely internal location of multiple vesicles.
Source data are provided as a Source Data file.

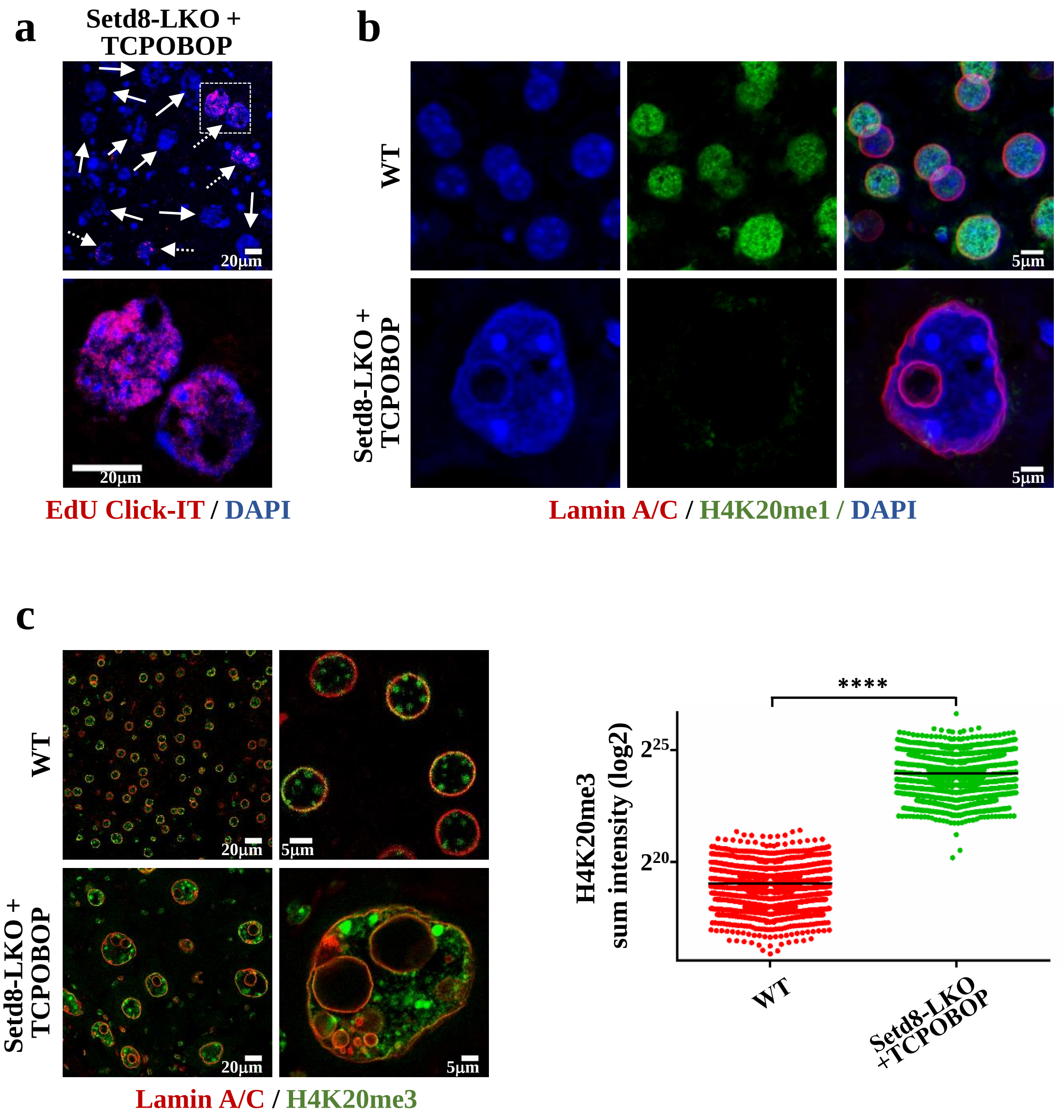

Supplementary Fig. 4

Supplementary Fig. 4

DNA replication activity and H4K20 methylation dynamics in TCPOBOP-treated Setd8-LKO hepatocytes.

a, Fluorescence Click-IT images of liver sections from mice pulse labeled (2 hours) by EdU, showing DNA replication activity in a fraction of TCPOBOP-treated Setd8-LKO hepatocytes 24 hours after treatment. Panel at the bottom, magnified image of the selected area. Dashed arrows indicate positively stained cells, solid arrows indicate cells that did not incorporate EdU into DNA.

b-c, Immunostaining with antibodies recognizing monomethylated **(b)** and trimethylated H4K20 **(c)**. The right panel of **(c)** shows measurements of $H4K20Me_3$ pixel intensities in individual cells. Measurements were from n=2500 wild type and n=2620 TCPOBOP-treated Setd8-LKO hepatocytes. Note the loss of $H4K20Me_1$ and the highly increased $H4K20Me_3$ levels in Setd8-LKO hepatocytes, indicating up-methylation of existing monomethylated histone H4. Data analyses were performed by Student's *t* test. Data are presented as median values (black line). ****p-value<0.0001.

Source data are provided as a Source Data file.

a

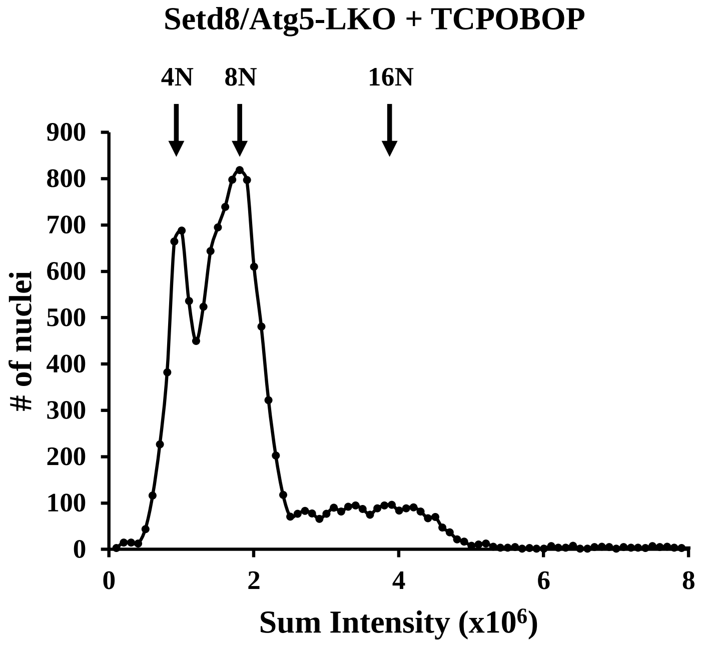

b

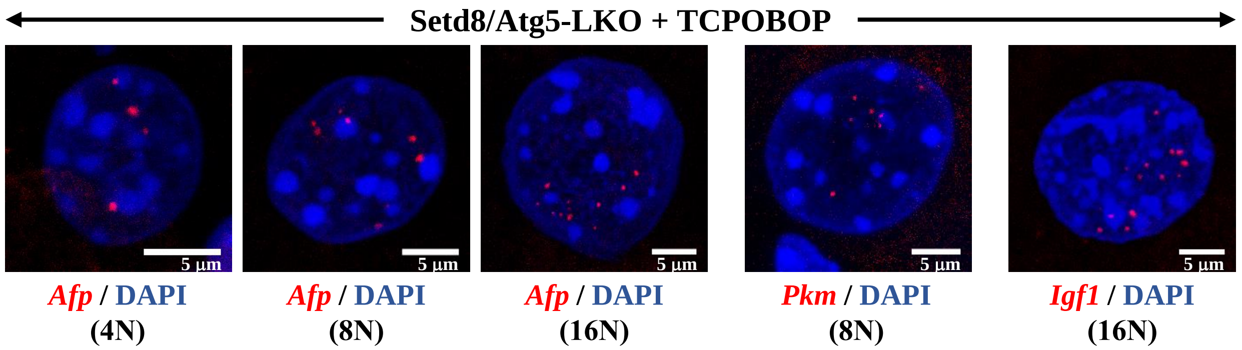

Supplementary Fig. 5

Supplementary Fig. 5

Limited polyploidy in Setd8/Atg5 double KO hepatocytes.
a, Estimation of nuclear DNA content in the nuclei of TCPOBOP-treated Setd8/Atg5-LKO livers (n=11771) by propidium iodide (PI) staining. The graph shows high-content microscopy (HCM) measurements of the PI staining intensity in individual cells. Arrows indicate the chromosomal ploidy of the peaks. The percentage of the cells corresponding to the peak areas were as follows: 4N= 26.8%; 8N=58%; 16N=15.2%.
b, Representative DNA FISH images (red) with probes spanning *Afp* (chr5), *Igf1* (chr10) and *Pkm* (chr9) genes.
Note the lack of 2N hepatocytes and polyploidy up to 16N DNA content by both assays.
Source data are provided as a Source Data file.

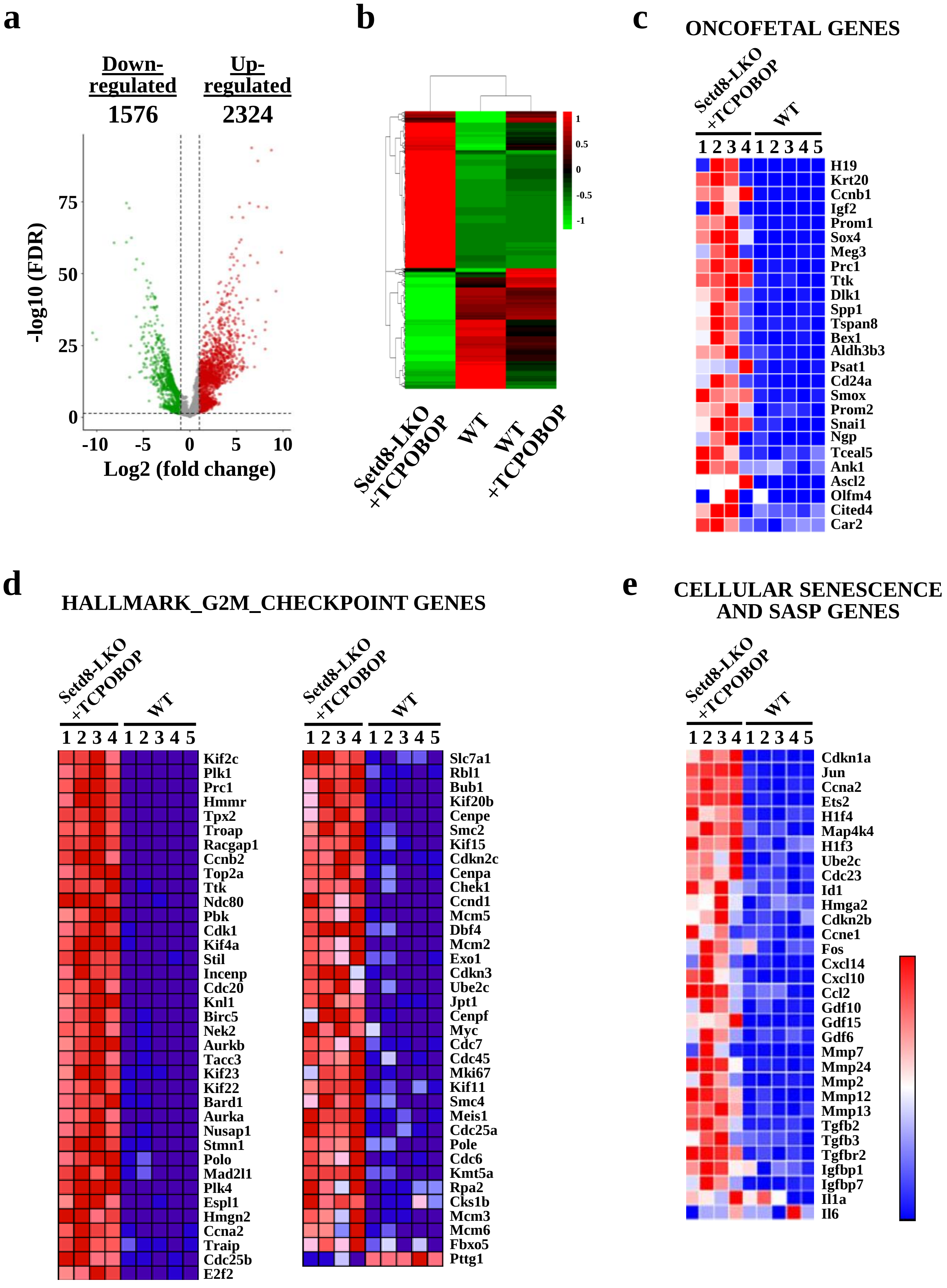

Supplementary Fig. 6

Supplementary Fig. 6

Analysis of global gene expression patterns in wild type and Setd8-LKO hepatocytes.
a, Volcano-plot of differentially expressed genes between wild type and TCPOBOP-treated Setd8-LKO hepatocytes. **b,** Hierarchical clustering analysis and corresponding heatmap of the differentially expressed genes in wild type, TCPOBOP-treated wild type and TCPOBOP-treated Setd8-LKO hepatocytes. Color bar values show log2 (fold change). Note the distinct profile of the latter, demonstrating Setd8 deficiency-dependent changes. **c-e,** Heatmap analysis of the normalized RPKM values (raw z-scored, variable units) of gene signatures representing hepatic oncofetal genes **(c)**, G2/M checkpoint genes **(d)** and senescence-specific genes including SASP genes **(e)**.

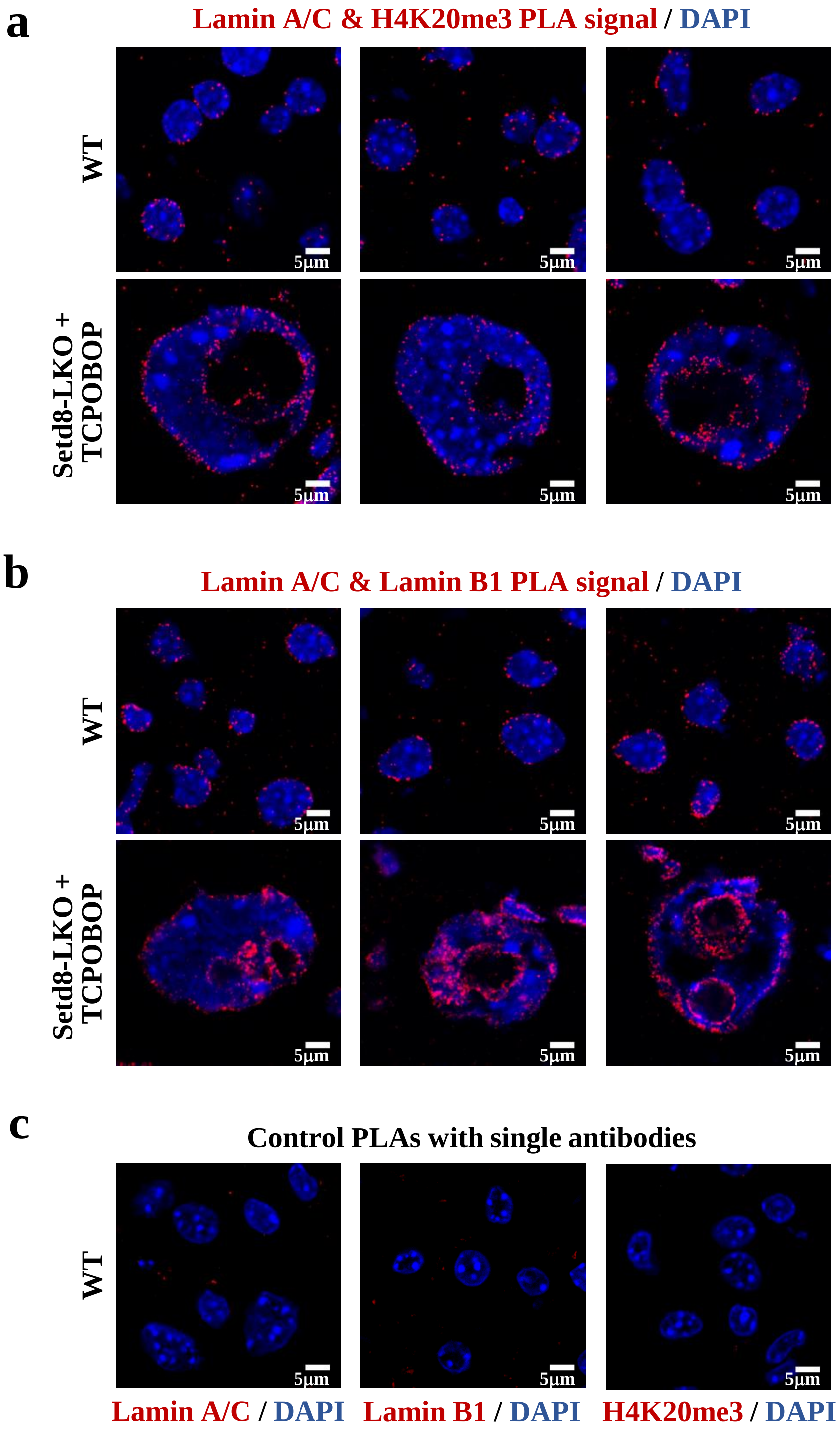

Supplementary Fig. 7

Supplementary Fig. 7

***In situ* detection of H4K20Me$_3$-modified nucleosomes associated with nuclear lamina.**

a, PLA images in liver sections from 3 different wild type and TCPOBOP-treated Setd8-LKO mice using antibodies recognizing H4K20Me$_3$ and Lamin A/C. Note the positive PLA signal at both nuclear envelope and internalized vesicle membranes. **b,** Positive control PLA reaction using Lamin A/C and Lamin B1 antibodies detecting physical proximity of Lamin A/C and Lamin B polymers at the nuclear envelope and at the endonucleotic vesicle membranes. **c,** Negative control PLA reaction with individual antibodies.

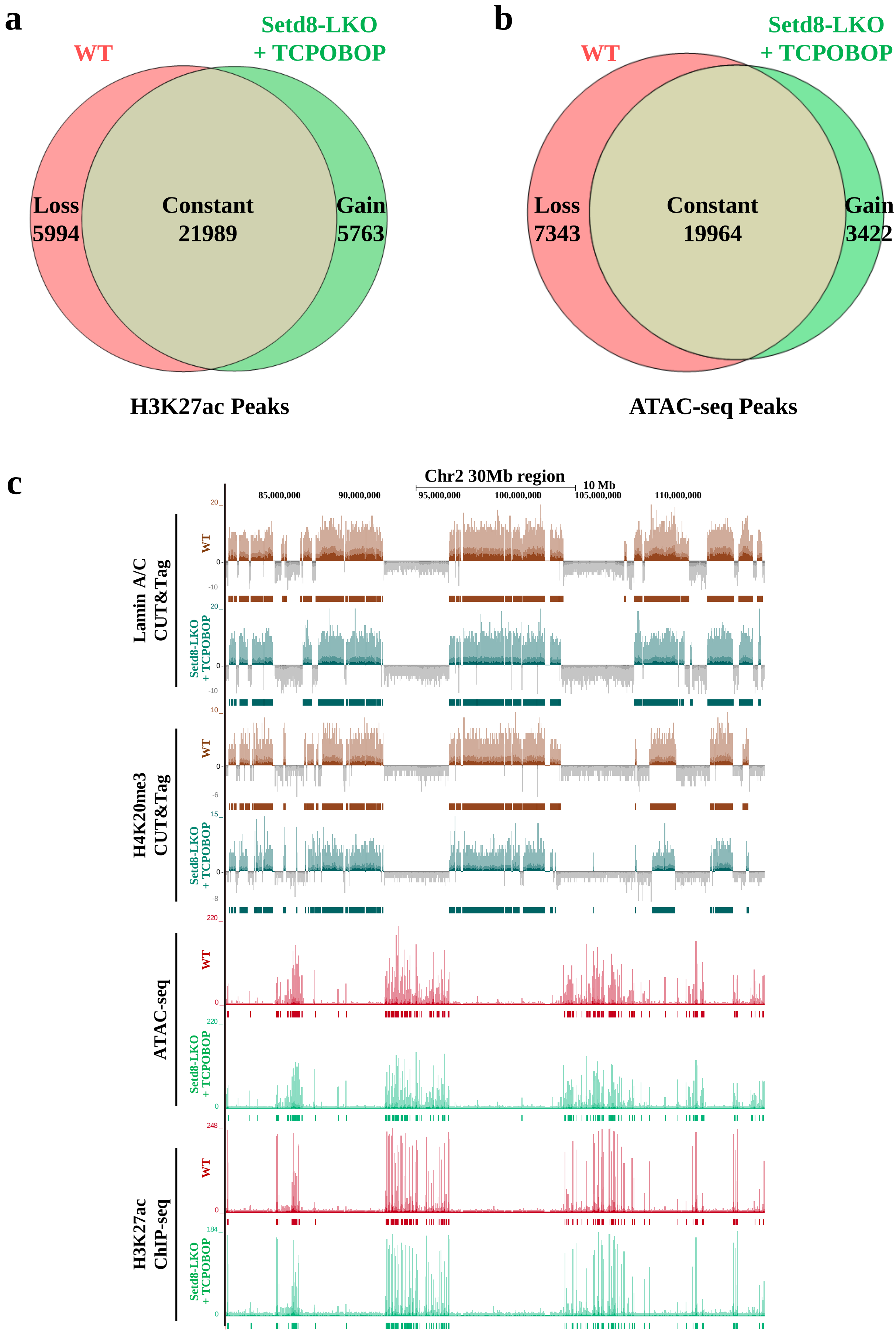

Supplementary Fig. 8

Supplementary Fig. 8

Analysis of H3K27ac nucleosome-containing and transposase-accessible, open euchromatin genomic areas in Setd8-LKO hepatocytes.
a-b, Venn diagram comparing H3K27ac ChIP-seq peaks **(a)** and ATAC-seq peaks **(b)** in wild type and TCPOBOP-treated Setd8-LKO livers. "Loss" indicates peaks present only in wild type livers, "Gain" indicates new peaks in Setd8-LKO livers. "Constant" correspond to peaks found in both, wild type and Setd8-LKO livers. **c,** Combined UCSC Genome Browser profile in 30 Mb regions of chromosome 2, showing LADs, $H4K20Me_3$-modified heterochromatin domains and euchromatin domains with high density of ATAC-seq and H3K27ac ChIP-seq reads. Note the high-level preservation of the tandemly organized euchromatin and heterochromatin domains in hyperploid Setd8-LKO hepatocytes.

a

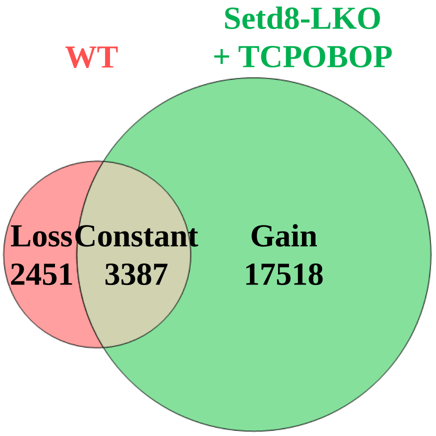

CTCF Peaks

b

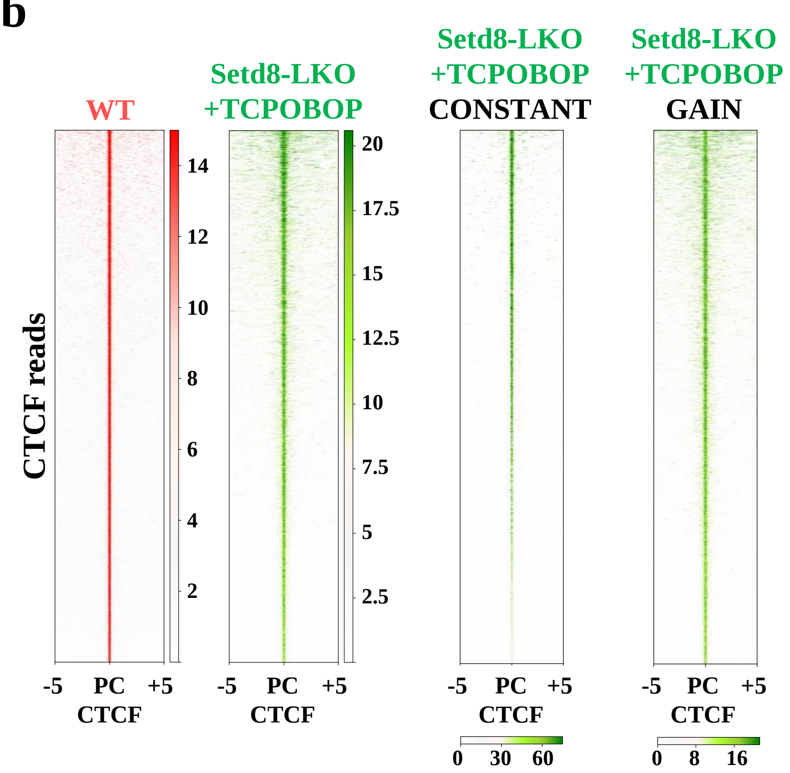

c

Top CTCF Peak Motifs

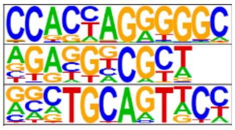

	WT all	P-value	% of Targets
1		1e-1187	68.13
2		1e-205	29.68
3		1e-110	15.42

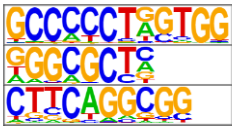

	Setd8-LKO Gain	P-value	% of Targets
1		1e-659	27.36
2		1e-97	28.06
3		1e-64	31.99

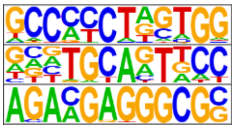

	WT Loss	P-value	% of Targets
1		1e-685	75.12
2		1e-101	27.17
3		1e- 39	3.93

Supplementary Fig. 9

Supplementary Fig. 9

Genome-wide profile of CTCF binding in Setd8-LKO cells.
a, Venn diagram showing the overlap of CTCF ChIP-seq peaks between wild type and Setd8-LKO mice 24 hours after TCPOBOP treatment. “Loss” indicates peaks present only in wild type livers, “Gain” indicates new peaks in Setd8-LKO livers, while “Constant” correspond to peaks found in both the wild type and the Setd8-LKO livers. **b,** Binding intensity heatmaps showing the distribution of genomic occupancy of CTCF in 5 kb downstream to 5 kb upstream areas from the CTCF peak center (PC, base pair showing the highest CTCF read pileup) in wild type and TCPOBOP-treated Setd8-LKO livers. The depicted regions were ranked by decreasing CTCF binding strength, as measured by the normalized fold enrichment of reads under CTCF peaks to the respective input regions. Panels at right show analyses restricted to “Constant” peaks and “Gain” peaks. Note the different scales in the color bars. Note the lower binding strength and the increased number of reads outside the PC of “Gain” peaks.
c, Sequence logos of the binding motifs identified under CTCF peaks by de novo motif search. The percent of targets with the canonical CTCF motif [38] are indicated in red color.

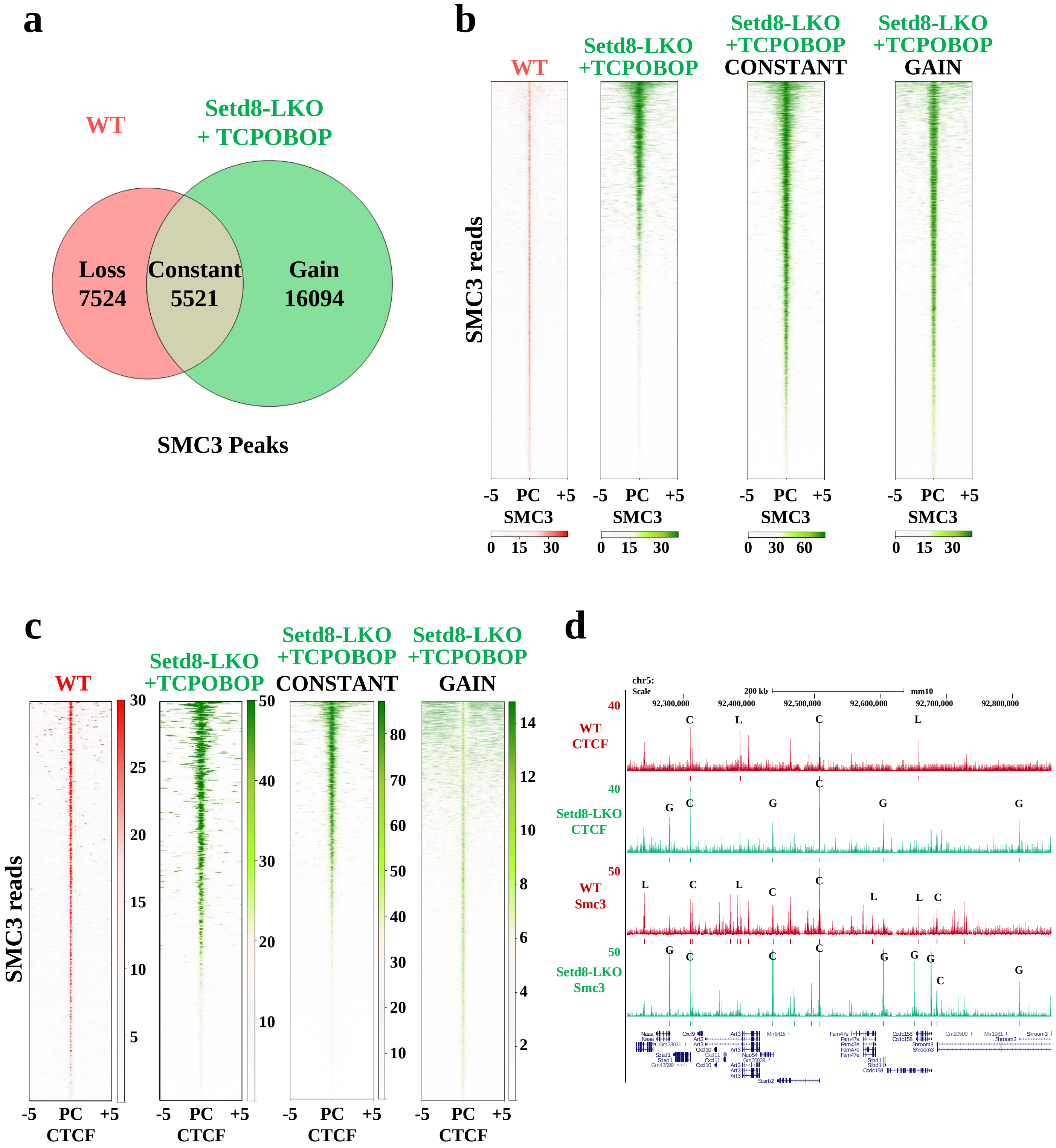

Supplementary Fig. 10

Supplementary Fig. 10

Genome-wide profile of Smc3 binding in Setd8-LKO cells

a, Venn diagram showing the overlap of Smc3 ChIP-seq peaks between wild type and Setd8-LKO mice 24 hours after TCPOBOP treatment. “Loss” indicates peaks present only in wild type livers, “Gain” indicates new peaks in Setd8-LKO livers, while “Constant” correspond to peaks found in both the wild type and the Setd8-LKO livers. **b,** Binding intensity heatmaps showing the distribution of genomic occupancy of Smc3. Heatmaps feature Smc3 reads around called Smc3 peak centers, ranked by decreasing signal strength. **c,** Binding intensity heatmaps of Smc3 reads in wild type and Setd8-LKO livers relative to the center of CTCF peaks identified in wild type or Setd8-LKO mice. The two panels at right show the distribution of “Constant” and “Gain” Smc3 peaks. Note the differences in the color bars indicating relative signal strength. Note the lower binding strength of “Gain” reads compared to “Constant” ones, and their low level of accumulation near CTCF locations. **d,** UCSC Genome Browser profile in 600 kb region of chromosome 5, showing examples for the Loss (L), Gain (G) and Constant (C) categories of CTCF and Smc3 binding profiles in wild type or Setd8-LKO livers.
