# Supplementary material for: Endonucleosis mediates internalization of cytoplasm into the nucleus
Source: Nat Commun. 2024 Jul 11;15:5843. doi: 10.1038/s41467-024-50259-3 (PMC11239883; doi:10.1038/s41467-024-50259-3)
Supplement: Supplementary file 3 — Reporting Summary [file 41467_2024_50259_MOESM3_ESM.pdf]

nature portfolio

Corresponding author(s): Iannis Talianidis

Last updated by author(s): Jul 2, 2024

Reporting Summary

Nature Portfolio wishes to improve the reproducibility of the work that we publish. This form provides structure for consistency and transparency in reporting. For further information on Nature Portfolio policies, see our Editorial Policies and the Editorial Policy Checklist.

Statistics

For all statistical analyses, confirm that the following items are present in the figure legend, table legend, main text, or Methods section.

n/a	Confirmed	
☐	☒	The exact sample size (*n*) for each experimental group/condition, given as a discrete number and unit of measurement
☐	☒	A statement on whether measurements were taken from distinct samples or whether the same sample was measured repeatedly
☐	☒	The statistical test(s) used AND whether they are one- or two-sided<br>*Only common tests should be described solely by name; describe more complex techniques in the Methods section.*
☒	☐	A description of all covariates tested
☒	☐	A description of any assumptions or corrections, such as tests of normality and adjustment for multiple comparisons
☐	☒	A full description of the statistical parameters including central tendency (e.g. means) or other basic estimates (e.g. regression coefficient) AND variation (e.g. standard deviation) or associated estimates of uncertainty (e.g. confidence intervals)
☐	☒	For null hypothesis testing, the test statistic (e.g. *F*, *t*, *r*) with confidence intervals, effect sizes, degrees of freedom and *P* value noted<br>*Give P values as exact values whenever suitable.*
☒	☐	For Bayesian analysis, information on the choice of priors and Markov chain Monte Carlo settings
☒	☐	For hierarchical and complex designs, identification of the appropriate level for tests and full reporting of outcomes
☒	☐	Estimates of effect sizes (e.g. Cohen's *d*, Pearson's *r*), indicating how they were calculated

Our web collection on statistics for biologists contains articles on many of the points above.

Software and code

Policy information about availability of computer code

Data collection	Data collected by Operetta High Content Screening Microscope (HCSM) in wide field were analyzed using Harmony software 4.1 with PhenoLOGIC (PerkinElmer) or the open source software FIJI/Image J
Data analysis	GraphPad Prism 6-9 was used to generate graphs and perform statistical analysis.<br>RNA-seq data were analyzed by using the Bioconductor package MretaseqR (Ref. 48). ChIP-seq and CUT&Tag data quality were assessed by FastQC (https://www.bioinformatics.babraham.ac.uk/projects/fastqc) as describe in the Methods section.

For manuscripts utilizing custom algorithms or software that are central to the research but not yet described in published literature, software must be made available to editors and reviewers. We strongly encourage code deposition in a community repository (e.g. GitHub). See the Nature Portfolio guidelines for submitting code & software for further information.

Data

Policy information about availability of data

All manuscripts must include a data availability statement. This statement should provide the following information, where applicable:

- Accession codes, unique identifiers, or web links for publicly available datasets
- A description of any restrictions on data availability
- For clinical datasets or third party data, please ensure that the statement adheres to our policy

RNA-seq, ChIP-seq, ATAC-seq and CUT&Tag data are available at the Gene Expression Omnibus (GEO) under accession number GSE242719

The Raw data from High content Microscopy measurements are available at the Supplementary Information.

Research involving human participants, their data, or biological material

Policy information about studies with human participants or human data. See also policy information about sex, gender (identity/presentation), and sexual orientation and race, ethnicity and racism.

Reporting on sex and gender	N/A
Reporting on race, ethnicity, or other socially relevant groupings	N/A
Population characteristics	N/A
Recruitment	N/A
Ethics oversight	N/A

Note that full information on the approval of the study protocol must also be provided in the manuscript.

Field-specific reporting

Please select the one below that is the best fit for your research. If you are not sure, read the appropriate sections before making your selection.

[x] Life sciences [ ] Behavioural & social sciences [ ] Ecological, evolutionary & environmental sciences

For a reference copy of the document with all sections, see nature.com/documents/nr-reporting-summary-flat.pdf

Life sciences study design

All studies must disclose on these points even when the disclosure is negative.

Sample size	Sample size was determined empirically based on similar studies with Setd8-LKO mice (Refs. 13 and 14). No statistical method was used to predetermine sample size. As a rule, all of the imaging-based assays were performed at least 2 different dates with 3 different biological replicates (mice) each time and at least 8-10 technical replicates (sections) each time. RNA-seq analyses involved RNAs from 4 and 5 biological replicates collected at the same date. ChIP-seq and CUT&Tag assays involved at least 2 biological replicates.
Data exclusions	No data was excluded from the analyses.
Replication	No attempts of replication failed. All of the data were replicated in multiple biological replicates and at different dates as described above.
Randomization	All experiments were performed in randomly chosen age-matched male mice. We compared mice with different genomic backgrounds and mice mock treated (with DMSO) or treated with 1,4-bis-[2-(3,5,-dichloropyridyloxy)] benzene (TCPOBOP)
Blinding	No blinding was used in this study.

Reporting for specific materials, systems and methods

We require information from authors about some types of materials, experimental systems and methods used in many studies. Here, indicate whether each material, system or method listed is relevant to your study. If you are not sure if a list item applies to your research, read the appropriate section before selecting a response.

Materials & experimental systems

n/a	Involved in the study	
[ ]	[x]	Antibodies
[x]	[ ]	Eukaryotic cell lines
[x]	[ ]	Palaeontology and archaeology
[ ]	[x]	Animals and other organisms
[x]	[ ]	Clinical data
[x]	[ ]	Dual use research of concern
[x]	[ ]	Plants

Methods

n/a	Involved in the study	
[ ]	[x]	ChIP-seq
[x]	[ ]	Flow cytometry
[x]	[ ]	MRI-based neuroimaging

Antibodies

Antibodies used	Antibodies were used for Immunofluorescence staining (IF), CUT&Tag or Chromatin Immunoprecipitation (ChIP) from the following

vendors:
Cell Signaling technologies:
Mouse anti-Lamin A/C (4C11), #4777, RRID:AB_10545756 (IF: 1:100, CUT&Tag)
Mouse anti-α-Tubulin (DM1A), #3873, RRID:AB_1904178 (IF: 1:400)
Rabbit anti-FANCD2 (D5L5X), #16323, RRID:AB_2798761 (IF: 1:100)
Rat anti-RPA32/RPA2 (4E4), #2208, RRID:AB_2238543 (IF: 1:50)
Rabbit anti-Phospho-Histone H2A.X (Ser139) (20E3), #9718, RRID:AB_2118009 (IF: 1:200)
Goat anti-mouse IgG (H+L), F(ab')2 Fragment (Alexa Fluor® 594 Conjugate), #8890, RRID:AB_2714182 (IF: 1:500)
Goat anti-rabbit IgG (H+L), F(ab')2 Fragment (Alexa Fluor® 488 Conjugate), #4412, RRID:AB_1904025 (IF: 1:500)
Goat anti-rabbit IgG (H+L), F(ab')2 Fragment (Alexa Fluor® 594 Conjugate), #8889, RRID:AB_2716249 (IF: 1:500)
Goat anti-mouse IgG (H+L), F(ab')2 Fragment (Alexa Fluor® 488 Conjugate), #4408, RRID:AB_10694704 (IF: 1:500)
Rabbit anti-NUP98 (C39A3), #2598, RRID:AB_2267700 (IF: 1:50)
Rabbit anti-PDI (C81H6), #3501, RRID:AB_2156433 (IF: 1:50)
Rabbit anti-ERp72 (D70D12) XP®, #5033, RRID:AB_10622112 (IF: 1:50)

From Abcam:
Rabbit anti-LaminB1, ab16048, RRID:AB_443298 (IF: 1:100)
Rabbit anti-SUN2 (EPR6557), ab124916, RRID:AB_10972497 (IF: 1:100)
Rabbit anti-SMC3, ab9263, RRID:AB_307122 (IF: 1:800, ChIP)
Rabbit anti-Histone H4 (tri methyl K20), ab9053, RRID:AB_306969 (IF: 1:200, IF and CUT&Tag)
Rabbit anti-Histone H4 (mono methyl K20), ab9051, RRID:AB_306967 (IF: 1:200)
Rabbit anti-Histone H3 (tri methyl K9), ab8898, RRID:AB_306848 (IF: 1:200)
Rabbit anti-Histone H3 (acetyl K27), ab4729, RRID:AB_2118291 (ChIP:1:100)
Rabbit anti-Ki67, ab15580, RRID:AB_443209 (IF: 1:200)
Rabbit anti-HMGB1, ab18256, RRID:AB_444360 (IF: 1:200)
Goat Anti-Guinea pig IgG H&L (Alexa Fluor® 647), ab150187, RRID:AB_2827756 (IF: 1:200)

From Novus Biologicals:
Rabbit anti-Calnexin, NB100-1974, RRID:AB_10001873 (IF: 1:50)

From BD Biosciences:
Mouse anti-E-cadherin (clone 36), 610181, RRID:AB_397580 (IF: 1:200)

From Santa Cruz Biotechnologies:
Rabbit anti-HNF4α (H-171), sc-8987, RRID:AB_2116913 (IF: 1:100)

From EMD Millipore:
Rabbit anti-CTCF, 07-729, RRID:AB_441965 (ChIP)

From Progen:
Guinea pig anti-p62/SQSTM1 (C-terminus), GP62-C, RRID:AB_2687531 (IF: 1:100)

From Proteintech:
Rabbit anti-Albumin, 16475-1-AP, RRID:AB_2242567 (IF: 1:50)
Rabbit anti-VAP-A, 15275-1-AP, RRID:AB_2256991 (IF: 1:50)

From Jackson Immuno Research Laboratory:
Alexa Fluor® 594 AffiniPure Donkey Anti-Rat IgG (H+L), 712-585-153, RRID:AB_2340689 (IF: 1:200)

From Thermo Fisher Scientific:
Rabbit anti-Histone Macro-H2A.1 Recombinant (RM248), MA5-24696, RRID:AB_2661883 (IF: 1:200)

Validation

Antibodies were validated by the manufacturers and the RRID database. RRID numbers are indicated above.

Animals and other research organisms

Policy information about studies involving animals; ARRIVE guidelines recommended for reporting animal research, and Sex and Gender in Research

Laboratory animals

C57Bl6 mice were used in this study. In experiments with Atg5-KO studies B6.129S mice were mixed with Transgenic mice in C57Bl6 strain. The mice were used at age between 45 and 110 days after birth.
Source of mice: Setd8lox/lox-AlbCre mice were generated in house (Ref. 15). ob/ob (B6.Cg-Lepob/J, JAX stock 000632) mice was from Jackson Laboratory. GFP-LC3 transgenic mice (RBRC00806) and Atg5 floxed (B6.129S-Atg5 [tm1Myok]) mice were from RIKEN BioResource Center and obtained from Georgios Chamilos (Univ. of Crete).

Wild animals	This study did not involve wild animals.
Reporting on sex	Only male mice were used in this study.
Field-collected samples	This study did not involve field-collected samples.
Ethics oversight	All animal experiments were approved by the Ethical Review Board of IMBB-FORTH and the Animal Ethics Committee of the Prefecture of Crete and were performed in accordance with the respective national and European Union regulations.

Note that full information on the approval of the study protocol must also be provided in the manuscript.

Plants

Seed stocks	N/A
Novel plant genotypes	N/A
Authentication	N/A

ChIP-seq

Data deposition

☒ Confirm that both raw and final processed data have been deposited in a public database such as GEO.

☒ Confirm that you have deposited or provided access to graph files (e.g. BED files) for the called peaks.

Data access links
May remain private before publication.

RNA-seq, ChIP-seq, ATAC-seq and CUT&Tag data are available at the Gene Expression Omnibus (GEO) under accession number GSE242719

Files in database submission

GSM7767557 Setd8-LKO1, TCPOBOP 24h, Mouse liver nuclei, ATAC-seq
GSM7767558 Setd8-LKO2, TCPOBOP 24h, Mouse liver nuclei, ATAC-seq
GSM7767559 Setd8-LKO3, TCPOBOP 24h, Mouse liver nuclei, ATAC-seq
GSM7767560 Setd8-LKO4, TCPOBOP 24h, Mouse liver nuclei, ATAC-seq
GSM7767561 Setd8-LKO5, TCPOBOP 24h, Mouse liver nuclei, ATAC-seq
GSM7767562 WT1, untreated, Mouse liver nuclei, ATAC-seq
GSM7767563 WT2, untreated, Mouse liver nuclei, ATAC-seq
GSM7767564 WT3, untreated, Mouse liver nuclei, ATAC-seq
GSM7767565 Setd8-LKO1, TCPOBOP 24h, H3K27ac, ChIP-seq
GSM7767566 Setd8-LKO2, TCPOBOP 24h, H3K27ac, ChIP-seq
GSM7767567 Setd8-LKO3,TCPOBOP 24h , H3K27ac, ChIP-seq
GSM7767568 Setd8-LKO1, TCPOBOP 24h, CTCF, ChIP-seq
GSM7767569 Setd8-LKO2, TCPOBOP 24h, CTCF, ChIP-seq
GSM7767570 Setd8-LKO1, TCPOBOP 24h, SMC3, ChIP-seq
GSM7767571 Setd8-LKO2, TCPOBOP 24h, SMC3. ChIP-seq
GSM7767572 Setd8-LKO1, TCPOBOP, input, ChIP-seq
GSM7767573 Setd8-LKO2,TCPOBOP, input, ChIP-seq
GSM7767574 WT1, untreated, H3K27ac, ChIP-seq
GSM7767575 WT2, untreated, H3K27ac, ChIP-seq
GSM7767576 WT3, untreated, H3K27ac, ChIP-seq
GSM7767577 WT1 untreated, CTCF, ChIP-seq
GSM7767578 WT2 untreated, CTCF, ChIP-seq
GSM7767579 WT3 untreated, CTCF, ChIP-seq
GSM7767580 WT4 untreated, CTCF, ChIP-seq
GSM7767581 WT1 untreated, SMC3, ChIP-seq
GSM7767582 WT2 untreated, SMC3, ChIP-seq
GSM7767583 WT3 untreated, SMC3, ChIP-seq
GSM7767584 WTuntr1, input, ChIP-seq
GSM7767585 WTuntr2, input, ChIP-seq
GSM7767586 Setd8-LKO1, TCPOBOP 24h, LaminA/C, Mouse liver nuclei, CUT&TAG-seq
GSM7767587 Setd8-LKO2, TCPOBOP 24h, LaminA/C, Mouse liver nuclei, CUT&TAG-seq
GSM7767588 Setd8-LKO3, TCPOBOP 24h, LaminA/C, Mouse liver nuclei, CUT&TAG-seq
GSM7767589 Setd8-LKO4, TCPOBOP 24h, LaminA/C, Mouse liver nuclei, CUT&TAG-seq
GSM7767590 Setd8-LKO1, TCPOBOP 24h, H4K20me3, Mouse liver nuclei, CUT&TAG-seq
GSM7767591 Setd8-LKO2, TCPOBOP 24h, H4K20me3, Mouse liver nuclei, CUT&TAG-seq
GSM7767592 WT1, untreated, LaminA/C, Mouse liver nuclei, CUT&TAG-seq
GSM7767593 WT2, untreated, LaminA/C, Mouse liver nuclei, CUT&TAG-seq
GSM7767594 WT1, untreated, LaminA/C, Mouse liver nuclei, CUT&TAG-seq NGS-2147
GSM7767595 WT2, untreated, LaminA/C, Mouse liver nuclei, CUT&TAG-seq NGS-2148

GSM7767596	WT1, untreated, H4K20me3, Mouse liver nuclei, CUT&TAG-seq
GSM7767597	WT2, untreated, H4K20me3, Mouse liver nuclei, CUT&TAG-seq
GSM7767598	Setd8-LKO1, TCPOBOP, RNA-seq
GSM7767599	Setd8-LKO2, TCPOBOP, RNA-seq
GSM7767600	Setd8-LKO3, TCPOBOP, RNA-seq
GSM7767601	Setd8-LKO4, TCPOBOP, RNA-seq
GSM7767602	WT1, untreated, RNA-seq
GSM7767603	WT2, untreated, RNA-seq
GSM7767604	WT3, untreated, RNA-seq
GSM7767605	WT4, untreated, RNA-seq
GSM7767606	WT5, untreated, RNA-seq
GSM7767607	WT1, TCPOBOP, 24h, RNA-seq
GSM7767608	WT2, TCPOBOP, 24h, RNA-seq
GSM7767609	WT3, TCPOBOP, 24h, RNA-seq
GSM7767610	WT4, TCPOBOP, 24h, RNA-seq
GSM7767611	WT5, TCPOBOP, 24h, RNA-seq

Genome browser session
(e.g. UCSC)

Provide a link to an anonymized genome browser session for "Initial submission" and "Revised version" documents only, to enable peer review. Write "no longer applicable" for "Final submission" documents.

Methodology

Replicates

For RNA-seq experiments at least 4 biological replicates were used.
For ChIP-seq and CUT&Tag experiments at least 2 biological replicates were used.

Sequencing depth

Average Sequencing depth for RNAseq: 40 million reads
Average Sequencing depth for ChIP-seq: 30 million reads
Average Sequencing depth for CUT&Tag: 10 million reads

Antibodies

The antibodies used fot ChIP-seq and CUT&Tag are indicated in the Antibody list above.

Peak calling parameters

Peak calling for ChIP data was performed by MACS2 (2.2.7.1) with default parameters and pvalue cutoff 1.00e-13. For CUT&Tag we used SICER2 (ref 46) with default parameters.

Data quality

Quality check for sequencing reads was performed by FastQC

Software

The following software tools were used: Trimmomatic (version 0.39); samtools version 1.10; deeptools (version 3.3.2); MACS2 (2.2.7.1); SICER2; HOMER45; computeMatrix.
